# The detection of great crested newts year round via environmental DNA analysis

**DOI:** 10.1186/s13104-017-2657-y

**Published:** 2017-07-26

**Authors:** Helen C. Rees, Claire A. Baker, David S. Gardner, Ben C. Maddison, Kevin C. Gough

**Affiliations:** 1 ADAS, SVMS, The University of Nottingham, Sutton Bonington Campus, Leicestershire, LE12 5RD UK; 2 School of Veterinary Medicine and Science, The University of Nottingham, Sutton Bonington Campus, Leicestershire, LE12 5RD UK

**Keywords:** Environmental DNA (eDNA), Great crested newts, Pond, Sampling season, Survey

## Abstract

**Objective:**

Analysis of environmental DNA (eDNA) is a method that has been used for the detection of various species within water bodies. The great crested newt (*Triturus cristatus*) has a short eDNA survey season (mid-April to June). Here we investigate whether this season could be extended into other months using the current methodology as stipulated by Natural England.

**Results:**

Here we present data to show that in monthly water samples taken from two ponds (March 2014–February 2015) we were able to detect great crested newt DNA in all months in at least one of the ponds. Similar levels of great crested newt eDNA (i.e. highly positive identification) were detected through the months of March–August, suggesting it may be possible to extend the current survey window. In order to determine how applicable these observations are for ponds throughout the rest of the UK, further work in multiple other ponds over multiple seasons is suggested. Nevertheless, the current work clearly demonstrates, in two ponds, the efficacy and reproducibility of eDNA detection for determining the presence of great crested newts.

**Electronic supplementary material:**

The online version of this article (doi:10.1186/s13104-017-2657-y) contains supplementary material, which is available to authorized users.

## Introduction

In the UK, great crested newts (*Triturus cristatus*) are protected under the Wildlife and Countryside Act 1981 (as amended) and the Conservation of Habitats and Species Regulations 2010. This means that developers and others involved in land-use change often have to survey for great crested newts (for more information see [1]). In 2014 Natural England sanctioned the use of eDNA analysis for the detection of the great crested newt and previous studies have evaluated its use as a monitoring technique for great crested newts in the UK as compared to field survey [1, 2].

eDNA within water bodies originates from sloughed cellular material or excretions/secretions and is likely to be degraded by the action of UV light and microbes within approximately 2–4 weeks [3–6], although under conditions of low temperature, UV-B, and alkalinity, larval eDNA from bullfrog (*Lithobates catesbeianus*) tadpoles remained detectible for up to 58 days (approximately 8 weeks) [6]. Thus, detection of species-specific eDNA in water bodies demonstrates the presence or recent presence of that species within that water body. Here we build on previous work [1] to evaluate the use of eDNA for the detection of great crested newts throughout the year.

## Methods

Two ponds in Boxworth (Cambridgeshire, UK) known to contain great crested newt populations were used to provide water samples for this study and were visually observed every month prior to sample collection (for description of the ponds please see Additional file 1). Water samples were collected monthly from March 2014 to February 2015 following the methods as stipulated by Natural England [7] (see Additional file 1).

DNA extractions and PCR were carried out as per the methods as stipulated by Natural England [7]. All samples were tested for PCR inhibitors prior to analysis for great crested newt eDNA (see Additional file 1). PCR plates were set up within a UV sterilised PCR cabinet, extraction blanks were also included (see Additional file 1). A sample was recorded as positive for great crested newts if one or more of the 12 PCR replicates were positive. Statistical analysis was conducted using mixed-effect models to determine the variation in eDNA scores through the year (see Additional file 2: Table S1). eDNA score was included as the response variate, ‘Month’ as a fixed effect and ‘Pond’ included as a random effect in the model. A separate analysis with ‘Season’ rather than ‘Month’ as a fixed effect was also specified a priori to determine if variation in eDNA score occurred monthly or by sampling season. These separate comparisons test whether eDNA score varies more than would be expected by chance within months of the year or within a pre-defined ‘season’ or survey window (April–June).

## Results and discussion

Over the 12-month period none of the samples were found to contain PCR inhibitors and great crested newt eDNA was detected in at least one pond in all months (Table 1; Additional file 3: Figure S1). This confirms data reported by Buxton et al. [8] which showed great crested newt eDNA to be present in at least one of eight replicated but naturally colonised ponds between March and September (samples were not collected between October and February) [8]. eDNA scores were high (median, 9 out of 12) during, and either side of, the current survey window, i.e. April–June and in March, July, and August which corresponds to when great crested newts were visually observed by our ecologists (Table 1). Outside of this period (i.e. September–February) eDNA scores fell significantly (median 3 out of 12) and great crested newts were not visually observed prior to sample collection. Based on these results, great crested newts were consistently detected in these two ponds from March through to August. This would therefore allow for significant extension to the current survey window.

**Table 1:** Real-time PCR amplification scores versus visual observations

	eDNA score 1	eDNA score 2	eDNA score 3	Visual observation
Pond 1 March	7/12	3/12	6/12	Present
Pond 2 March	11/12	11/12	12/12	Present
Pond 1 April	12/12	12/12	11/12	Present
Pond 2 April	12/12	12/12	11/12	Present
Pond 1 May	6/12	9/12	3/12	Present
Pond 2 May	11/12	9/12	9/12	Present
Pond 1 June	5/12	4/12	4/12	Present
Pond 2 June	12/12	11/12	12/12	Present
Pond 1 July	6/12	11/12	9/12	Present
Pond 2 July	12/12	12/12	12/12	Present
Pond 1 August	7/12	7/12	8/12	Present
Pond 2 August	12/12	12/12	11/12	Present
Pond 1 September	1/12	0/12	0/12	Absent
Pond 2 September	3/12	2/12	0/12	Absent
Pond 1 October	0/12	0/12	0/12	Absent
Pond 2 October	0/12	2/12	0/12	Absent
Pond 1 November	0/12	1/12	0/12	Absent
Pond 2 November	1/12	4/12	1/12	Absent
Pond 1 December	1/12	0/12	1/12	Absent
Pond 2 December	0/12	0/12	0/12	Absent
Pond 1 January	6/12	0/12	3/12	Absent
Pond 2 January	2/12	3/12	4/12	Absent
Pond 1 February	6/12	4/12	5/12	Present
Pond 2 February	2/12	0/12	0/12	Present

Real-time PCR amplification scores (numbers of individual PCRs positive out of a maximum of 12 replicates) for eDNA extracted from water samples taken monthly from two ponds over a year. eDNA was measured using PCR × 12 replicates for each of three separate PCR amplifications of a single eDNA extraction. Visual observations were made prior to collection of the water samples every month and great crested newts recorded as present or absent

It is known that great crested newts and their larvae often overwinter in water, therefore there can be some life stage of the great crested newt in water all year round [9]. The PCR amplification score profile (Additional file 3: Figure S1) appears in general to match the yearly life cycle of the great crested newt, and their known occupancy of the aquatic environment. The great crested newt breeding season peaks in mid-March to May and during this time great crested newts are likely to be present in ponds and pools [9]. This correlates with the high amplification scores between March and May observed in the present study. Once hatched the larvae live in these ponds until they develop into air-breathing juveniles after which they will begin to emerge from the ponds during August although they are known to overwinter in ponds [10]. Indeed there was no significant difference in eDNA amplification scores between those samples taken within the survey season (April–June) and those taken in March, July and August (Additional file 3: Figure S1). From the presented data this lifecycle stage would appear to produce samples with relatively high amplification scores up to and including August, after which they reduce in September. Adults and juveniles normally live on land and are thought to hibernate between October and February [9] and this correlates with the presented data showing that there is a significant difference between those samples taken within the season (April–June) and those taken between September and February (Additional file 3: Figure S1). These results would suggest that it might be possible to extend the survey season to include the months of March, July and August.

When considering the eDNA persistence, the detection of great crested newts during July and August, outside of the normal breeding season, is most likely due to great crested newt presence or recent presence and not residual eDNA signal left over from the breeding season. The warmer temperatures in June to August (average of 15.0–7.6 °C [11]) would be expected to degrade eDNA faster than the 58 days seen by Strickler et al. at 5 °C [6]. The presence of great crested newt eDNA in March is probably not that generalizable prior to the usual breeding season as it is likely to be heavily dependent on weather conditions and geographical location being suitable for early movement of great crested newt into ponds.

## Limitations

This study did not look to deviate from the accepted Natural England method [7], but future studies could look to optimise the sampling regimes. Similar analyses of additional ponds throughout the UK are required to demonstrate how applicable these observations are to the rest of the UK. In addition, analyses of samples taken over several seasons are required to demonstrate the reproducibility of this eDNA detection across years, which may present distinct breeding conditions for great crested newt.

## Additional files

**Additional file 1.** Additional information on the methodology employed in the study.

**Additional file 2: Table S1.** Table of the raw data analysed using Genstat v18, VSNi, Rothampstead, UK.

**Additional file 3: Figure S1.** Real-time PCR scores and statistical analysis for two ponds over a 12-month period.

## Abbreviation

eDNA: environmental DNA
