# Supplementary material for: The detection of great crested newts year round via environmental DNA analysis
Source: BMC Res Notes. 2017 Jul 26;10:327. doi: 10.1186/s13104-017-2657-y (PMC5530555; doi:10.1186/s13104-017-2657-y)
Supplement: Supplementary file 1 — Additional file 1. Additional information on the methodology employed in the study. [file 13104_2017_2657_MOESM1_ESM.docx]

**Additional File 1**

**Description of Ponds**

The ponds are situated within farmland laid to grass and the main aquatic plant within the ponds is *Potamogeton natans* (*Elodea canadensis*, *Potamogeton pectinatus* and *Myriophyllum spicatum* are also present in smaller numbers). The ponds have dimensions 11 m x 7 m x 1.65 m (length x width x depth) are approximately 3 m apart, and comprise a 10-20 cm clay loam base sediment layer and a nominal water body depth of 110 cm ± 10%. A full great crested newt survey was not carried out as great crested newt have been identified within these ponds each year for approximately 10 years. However, every month prior to sample collection the circumference of the ponds were walked and monitored for great crested newt presence a full survey was not carried out as great crested newts have been identified each year within these ponds for ~10 years.

**Water Sample Collection**

Twenty samples were taken from around the perimeter of the pond with a sterile sampling ladle, pooled into a sterile bag and mixed before transferring six lots of 15 mL into six 50 mL falcon tubes each containing 33.5 mL absolute ethanol (200 proof), and 1.5 mL 3M sodium acetate pH 5.2.

**DNA extraction**

DNA was recovered by centrifugation (5000g, 35 min, 6°C), the supernatant discarded, and the pellet air dried. Resulting pellets were extracted using the DNeasy Blood and Tissue kit (Qiagen) following the manufacturer’s instructions and finally resuspended in 200 µL of elution buffer. Extraction blanks consisting of tap water in place of pond water to test for cross-contamination were also included.

**PCR inhibition testing**

All extracted samples were subject to testing for PCR inhibitors prior to testing for great crested newts. PCR reactions were set up in a total volume of 25 µL consisting of: 3 µl of 0.08ng µl^-1^ plasmid DNA, 3 µL of extracted template DNA, 1 µL of each primer (0.4 µM), 1 µL of probe (0.1 µM), and 1x TaqMan® Environmental Master Mix 2.0 (containing AmpliTaq GOLD DNA polymerase, Life Technologies). The PCR included an initial incubation for 5 min at 50°C, then a 10 min denaturation step at 95°C, followed by 30 cycles of denaturation at 95°C for 30 s and annealing at 60°C for 1 min.

Extracted samples were analysed in duplicate and had an average Cq of 12.34 (Cqs ranged from 12.02 to 12.89). As a positive control a 5-fold dilution series of plasmid DNA from 0.08 ng µl^-1^ to 0.0032 ng µl^-1^ was used. Each positive control sample contained 3 µl of ultrapure water in place of extracted sample and was run in triplicate. The positive control which was equivalent to that spiked into extracted samples gave an average Cq of 12.65. Negative controls in triplicate contained 6 µL ddH_2_O in place of any DNA and were all found to be negative for amplification.

**Inhibition testing Primers and Probe**

Forward primer: InhibL 5’-GCAGCTGCAGGCTTACRGT-3’

Reverse primer: InhibR 5’-ACCGTTCAGGTCTCCTTCAG-3’

Probe: Inhib.probe 5’-CAACCGGCCATGGCTATGGA-3’; 5’-FAM; 3’-BHQ1

**GCN Primers and Probe**

Forward primer: TCCBL 5’-CGTAAACTACGGCTGACTAGTACGAA-3’

Reverse primer: TCCBR 5’-CCGATGTGTATGTAGATGCAAACA-3’

Probe: TCCB.probe 5’-CATCCACGCTAACGGAGCCTCGC-3’; 5’-FAM; 3’-BHQ1

The primers and probe have previously been validated (Biggs et al. 2014a; Rees et al. 2014a; Thomsen et al. 2012b) and their use for great crested newt eDNA detection has been sanctioned by Natural England in the UK.

**Real-time PCR**

PCR reactions were set up in a total volume of 25 µL consisting of: 3 µL of extracted template DNA, 1 µL of each primer (0.4 µM), 1 µL of probe (0.1 µM), and 1x TaqMan® Environmental Master Mix 2.0 (containing AmpliTaq GOLD DNA polymerase, Life Technologies). The PCR included an initial incubation for 5 min at 50°C, then a 10 min denaturation step at 95°C, followed by 55 cycles of denaturation at 95°C for 30 s and annealing at 60°C for 1 min.

Each 96-well plate contained: 12 replicates of up to six samples or extraction blanks to be analysed per plate (all samples were analysed in 12 replicates for each of three separate PCR amplifications for a single eDNA extraction); a 10-fold dilution series of great crested newt DNA as positive controls, 3 µL per quadruplicate replicates of 10^-1^ ng µl^-1^ to 10^-4^ ng µl^-1^; and a negative control again in quadruplicate with 3 µL ddH_2_O in place of the DNA.

All extraction blanks that were tested were negative. All positive and negative controls gave the expected results.

**Statistical Analysis**

To determine the variation in eDNA scores through the year, all scores (on a scale of 0 to 12) for the three PCR tests were incorporated into a Generalised Linear mixed Model, fit with a Binomial error distribution (response variate = eDNA score; Binomial total = 12) and a logit-link function for the fixed effects of either ‘Month’ or ‘Season’. ‘Pond’ was included as a random effect in the model. Data were analysed using Genstat v18, VSNi, Rothampstead, UK. ‘Season’ indicates current sampling period (as stipulated by Natural England). See also Table S1.
