# Supplementary material for: The detection of great crested newts year round via environmental DNA analysis
Source: BMC Res Notes. 2017 Jul 26;10:327. doi: 10.1186/s13104-017-2657-y (PMC5530555; doi:10.1186/s13104-017-2657-y)
Supplement: Supplementary file 2 — Additional file 2: Table S1. Table of the raw data analysed using Genstat v18, VSNi, Rothampstead, UK. [file 13104_2017_2657_MOESM2_ESM.docx]

**Table S1. Raw data for statistical analysis**

| Pond! | Month! | Season! | PCR! | eDNAscore | totalscore | Presentabsent! |
| --- | --- | --- | --- | --- | --- | --- |
| 0 | January | 1 | 2 | 0 | 12 | 0 |
| 0 | January | 1 | 3 | 3 | 12 | 1 |
| 0 | January | 1 | 1 | 6 | 12 | 1 |
| 0 | February | 1 | 2 | 4 | 12 | 1 |
| 0 | February | 1 | 3 | 5 | 12 | 1 |
| 0 | February | 1 | 1 | 6 | 12 | 1 |
| 0 | March | 1 | 2 | 3 | 12 | 1 |
| 0 | March | 1 | 3 | 6 | 12 | 1 |
| 0 | March | 1 | 1 | 7 | 12 | 1 |
| 0 | April | 0 | 3 | 11 | 12 | 1 |
| 0 | April | 0 | 1 | 12 | 12 | 1 |
| 0 | April | 0 | 2 | 12 | 12 | 1 |
| 0 | May | 0 | 3 | 3 | 12 | 1 |
| 0 | May | 0 | 1 | 6 | 12 | 1 |
| 0 | May | 0 | 2 | 9 | 12 | 1 |
| 0 | June | 0 | 2 | 4 | 12 | 1 |
| 0 | June | 0 | 3 | 4 | 12 | 1 |
| 0 | June | 0 | 1 | 5 | 12 | 1 |
| 0 | July | 1 | 1 | 6 | 12 | 1 |
| 0 | July | 1 | 3 | 9 | 12 | 1 |
| 0 | July | 1 | 2 | 11 | 12 | 1 |
| 0 | August | 1 | 1 | 7 | 12 | 1 |
| 0 | August | 1 | 2 | 7 | 12 | 1 |
| 0 | August | 1 | 3 | 8 | 12 | 1 |
| 0 | September | 1 | 2 | 0 | 12 | 0 |
| 0 | September | 1 | 3 | 0 | 12 | 0 |
| 0 | September | 1 | 1 | 1 | 12 | 1 |
| 0 | October | 1 | 1 | 0 | 12 | 0 |
| 0 | October | 1 | 2 | 0 | 12 | 0 |
| 0 | October | 1 | 3 | 0 | 12 | 0 |
| 0 | November | 1 | 1 | 0 | 12 | 0 |
| 0 | November | 1 | 3 | 0 | 12 | 0 |
| 0 | November | 1 | 2 | 1 | 12 | 1 |
| 0 | December | 1 | 2 | 0 | 12 | 0 |
| 0 | December | 1 | 1 | 1 | 12 | 1 |
| 0 | December | 1 | 3 | 1 | 12 | 1 |
| 1 | September | 1 | 1 | 0 | 12 | 0 |
| 1 | September | 1 | 2 | 0 | 12 | 0 |
| 1 | September | 1 | 3 | 0 | 12 | 0 |
| 1 | September | 1 | 2 | 0 | 12 | 0 |
| 1 | September | 1 | 3 | 0 | 12 | 0 |
| 1 | September | 1 | 1 | 0 | 12 | 0 |
| 1 | September | 1 | 3 | 0 | 12 | 0 |
| 1 | September | 1 | 3 | 0 | 12 | 0 |
| 1 | September | 1 | 1 | 1 | 12 | 1 |
| 1 | September | 1 | 3 | 1 | 12 | 1 |
| 1 | September | 1 | 1 | 2 | 12 | 1 |
| 1 | September | 1 | 1 | 2 | 12 | 1 |
| 1 | September | 1 | 2 | 2 | 12 | 1 |
| 1 | September | 1 | 2 | 2 | 12 | 1 |
| 1 | September | 1 | 2 | 3 | 12 | 1 |
| 1 | September | 1 | 1 | 3 | 12 | 1 |
| 1 | September | 1 | 3 | 4 | 12 | 1 |
| 1 | September | 1 | 2 | 4 | 12 | 1 |
| 1 | September | 1 | 2 | 9 | 12 | 1 |
| 1 | September | 1 | 3 | 9 | 12 | 1 |
| 1 | September | 1 | 3 | 11 | 12 | 1 |
| 1 | September | 1 | 3 | 11 | 12 | 1 |
| 1 | September | 1 | 2 | 11 | 12 | 1 |
| 1 | September | 1 | 1 | 11 | 12 | 1 |
| 1 | September | 1 | 2 | 11 | 12 | 1 |
| 1 | September | 1 | 1 | 11 | 12 | 1 |
| 1 | September | 1 | 1 | 12 | 12 | 1 |
| 1 | September | 1 | 2 | 12 | 12 | 1 |
| 1 | September | 1 | 1 | 12 | 12 | 1 |
| 1 | September | 1 | 2 | 12 | 12 | 1 |
| 1 | September | 1 | 1 | 12 | 12 | 1 |
| 1 | September | 1 | 2 | 12 | 12 | 1 |
| 1 | September | 1 | 3 | 12 | 12 | 1 |
| 1 | September | 1 | 1 | 12 | 12 | 1 |
| 1 | September | 1 | 3 | 12 | 12 | 1 |
| 1 | September | 1 | 3 | 12 | 12 | 1 |

Table of the raw data analysed using Genstat v18, VSNi, Rothampstead, UK.
