# Supplementary material for: The detection of great crested newts year round via environmental DNA analysis
Source: BMC Res Notes. 2017 Jul 26;10:327. doi: 10.1186/s13104-017-2657-y (PMC5530555; doi:10.1186/s13104-017-2657-y)
Supplement: Supplementary file 3 — Additional file 3: Figure S1. Real-time PCR scores and statistical analysis for two ponds over a 12-month period. [file 13104_2017_2657_MOESM3_ESM.docx]

**Figure S1:** Real-time PCR scores and statistical analysis for two ponds over a 12-month period.

The figure shows two comparisons to reflect statistical analysis of eDNA score with Month (P=0.31) as a fixed effect or with Season as the fixed effect (P=0.03). Dot-plot shows scores for each month by Pond, with horizontal line at median. ‘av S.E.’ is the standard error of the differences between means for all months/ponds, any points outside the range of the bar are significant at P<0.05. ‘SEASON’ indicates current sampling period (as stipulated by Natural England).
